# Blood Lead Levels and Risk Factors among Preschool Children in a Lead Polluted Area in Taizhou, China

**DOI:** 10.1155/2017/4934198

**Published:** 2017-03-29

**Authors:** Zhenyan Gao, Jia Cao, Jin Yan, Ju Wang, Shizhong Cai, Chonghuai Yan

**Affiliations:** ^1^Ministry of Education-Shanghai Key Laboratory of Children's Environmental Health, Xinhua Hospital, Shanghai Jiao Tong University School of Medicine, Shanghai, China; ^2^Children's Hospital of Soochow University, Suzhou, China

## Abstract

*Objective*. To determine the blood lead levels and identify related risk factors among preschool children in a lead polluted area (Taizhou, China) and provide theoretical support for prevention of lead pollution.* Methods*. A stratified-clustered-random sampling method was used to determine the survey sample. Blood lead levels were determined by the tungsten atomizer absorption spectrophotometer.* Results*. A total of 2,018 subjects (average age of 59 months; 1,087 boys and 931 girls) were included. The arithmetic mean, geometric mean, and median blood lead levels of the preschool children were 56.4 *μ*g/L, 48.9 *μ*g/L, and 46 *μ*g/L. A total of 8.8% children had blood lead levels >100 *μ*g/L and 43.9% had blood lead levels >50 *μ*g/L. Mother's education level, father's occupation, decorative tableware, exposure to makeup, and the residential floor were all risk factors for elevated blood lead levels (odds ratios of 1.42, 1.21, 1.11, 1.19, and 1.27, resp.), while hand washing before eating food was a protective factor (odds ratio of 0.88).* Conclusions*. The blood lead levels of preschool children in Taizhou were higher than in other areas in China and in developed countries. Therefore, policies ensuring lead-based industries are not placed in close proximity to residential areas are required.

## 1. Introduction

Lead is recognized as an environmental pollutant that is a major threat to public health [1–5]. Lead can contaminate soil, water, and air and is absorbed and spread through the blood, resulting in long-term accumulation in the organs [6]. As the lead uptake, absorption rates, and accumulation in children are generally higher than in adults, they are more susceptible to the effects of lead exposure [7]. Several studies indicate that lead exposure is negatively associated with intelligent quotient (IQ) levels [8, 9]. Lead also affects the hematopoietic, digestive, urinary [10], cardiovascular [11], endocrine, and immune systems, thereby eventually negatively impacting a child's growth [6, 12]. Other studies show that the lead concentration in the blood mediates brain volume and fine motor function [13] and that lead exposure in childhood predicts intellectual functioning in young adulthood [14]. Indeed, the specific injurious effects on human cells, including the changes in gene and protein expression changes, that occur in response to lead challenges have been documented [15].

The American Academy of Pediatrics defines lead poisoning as a blood lead level (BLL) of ≥100 *μ*g/L, which leads to harmful effects in the nervous, hematological, and urinary systems [16]. On the other hand, the National Toxicology Program (NTP) concluded that BLLs of <100 *μ*g/L were still associated with delayed puberty, poor cognitive performance, and a lower IQ in the United States [17]. Previous study showed that low level lead exposure (the median of BLL 20 *μ*g/L) had also impaired children's cognitive ability [9]. Indeed, another study showed that low level prenatal lead exposure effected the auditory recognition memory in 2-month-old infants [8]. While the American Center for Disease Control currently uses a BLL reference value of >50 *μ*g/L to indicate lead poisoning [18], there is no known BLL to date that has been shown to be too small to cause disease in children.

To reduce lead exposure, lead free gasoline was forcibly adopted by the Chinese Government on July 1, 2000. Since then, the prevalence of lead poisoning in children has decreased from 34% to approximately 24% [3]. Furthermore, when analyzing the epidemiological data, Li et al. found that BLLs declined in children aged 0–18 years from 1990 to 2012 [16]. By 2013, the BLLs of children aged 0–6 years in Hunan Province were only 20 *μ*g/L [19]. Similarly, the BLLs of children in Shanghai in 2014 were much lower than the earlier reported data [20]. This decline in BLLs appears to be associated with national efforts to decrease lead pollution, including the closure or merger of heavily polluting enterprises [21–23]. Despite this, many lead-polluting industries have migrated from large cities to middle-sized or small cities or to rural areas. Meanwhile, the production of lead-acid batteries has risen sharply because of their enhanced demand in the transport sector [3, 24]. Indeed, Taizhou, a coastal city of Zhejiang province, has experienced a significant expansion in lead smelting, battery production and recycling, e-waste recycling, metal processing, and production of lead-containing chemicals. Moreover, a substantial increase in the number of small family businesses that use lead-containing products has occurred in this region in recent years. As a result, despite the overall decline in children's BLLs in China over the past 10 years [16, 19, 23], children's exposure to lead is still common in Taizhou.

The aim of this study was to analyze the BLLs in children (aged 3–6 years) from a lead polluted area in China (Taizhou) and to reveal the major risk factors for BLLs in this region.

## 2. Subjects and Methods

### 2.1. Ethics Statement

This study was approved by the Medical Ethics Committee of Xinhua Hospital affiliated to Shanghai Jiao Tong University School of Medicine.

### 2.2. Subjects

This study was conducted in Taizhou City, Zhejiang province, China, from April 2013 to November 2013. A stratified-clustered-random sampling method was used in the survey. The Fengjiang and Pengjie districts were selected as the areas for investigation. Written informed consent was acquired for each child (from their parents). We defined BLLs of >100 *μ*g/L as elevated BLLs (EBLLs), based on the guidelines by the Medical Administration Department of the Ministry of Health of China in 2006. The 2012 American Center for Disease Control criteria for lead poisoning were used (i.e., a BLL of >50 *μ*g/L).

### 2.3. Questionnaire

A questionnaire was used to determine background information on the subjects, including the children's age, sex, physical growth indices, their parents' education and occupation, housing environment, residential history, and source of drinking water. The questions were selected based on our previous studies and literature research on the risk factors for lead poisoning in children.

### 2.4. Methods

A 3 mL venous blood sample was collected for each child by trained nurses. Whole blood was stored in vacutainer, with EDTA(Na2) used as an anticoagulant. Sample collection was performed in a clean room (i.e., without dust and smoke), which was far away from any source of lead. Before blood sampling, nurses and medical staff were required to wear white coats, cut their nails short, wash their hands with a 2% EDTA(Na2) tampon, and sterilize the skin with 75% alcohol. Children were required to clean their hand with soap before the blood was collected.

### 2.5. Quality Control

Blood samples were stored at 4°C before the lead analysis. The collection courses were accurate. The BLL was analyzed using atomic absorption spectrometry (PekinElmer Company 900Z USA). In order to ensure the quality of the results during lead detection, quality control was performed using a sample test. The laboratory protocol includes daily calibration with five standards (1–50 *μ*g/dL; agreement <5%) and standard reference materials (Contox, Kaulson Laboratories, Inc., NJ, USA) used to ensure the accuracy of the assay. Low and high lead levels of the standard blood were detected every day and all of the results were within the scope of the standard value. Low, medium, and high lead level controls were used for calibration starting from a standard reference material containing 50 *μ*g/dL. 50 *μ*L blood samples were added every time and ten times diluted. Analysis of each sample was performed in duplicate, and the mean of both values was determined. Every 50 measurements, a blank sample was analyzed to prevent between samples contamination. The limit of detection of the method (LOD) was 1 *μ*g/dL for BLL. No sample had a BLL less than the LOD.

### 2.6. Statistical Analysis

Sample characteristics were summarized by descriptive statistics. A threshold of 50 *μ*g/L or 100 *μ*g/L for BLLs was used to categorize BLLs into different groups. The distribution of BLL was not normal, after the logarithmic transformation. It was approximate normal distribution for the statistical analysis. Differences between groups were assessed using the Chi-square test to evaluate the prevalence of EBLLs and general linear model to evaluate BLLs among different groups. Bonferroni test was used as multiple comparison method among any two groups. Furthermore, logistic regression was used to estimate odds ratios (OR), 95% confidence intervals (CI), and *p* values. SAS software 9.2 (SAS Institute Inc., Cary, NC) was used for statistical analyses. A *p* value of <0.05 (two-tailed test) was considered statistically significant.

## 3. Results

The total number of subjects included was 2,018 (average age of 59 months, ranging from 20 to 96 months). The numbers of boys and girls were 1,087 and 931, respectively. BLLs ranged from 10.0 to 468.0 *μ*g/L, and the arithmetic mean, geometric mean, and median were 56.4 *μ*g/L, 48.9 *μ*g/L, and 46.0 *μ*g/L, respectively. A total of 8.8% (178/2018) of subjects had BLLs >100 *μ*g/L and 43.9% (886/2018) had BLLs >50 *μ*g/L. The main characteristics of the children included in the study are shown in Table 1.

The distribution of BLL was not normal, after the logarithmic transformation. It was approximate normal distribution for the statistical analysis. As shown in Table 2, significant differences in BLLs were observed with respect to the child's sex and age, their parents' education level and occupation, the family's economic situation, and their living environment. Boys had significantly higher BLLs than girls (*F* = 25.94; *p* < 0.05). In addition, the groups with BLLs ≥100 *μ*g/L and ≥50 *μ*g/L had significantly more boys than girls (*p* < 0.05). The BLLs of children from different age groups had different BLLs (*F* = 2.88; *p* < 0.05), but the multiple comparison (Bonferroni test) showed that this difference was not statistically significant in any two groups. Children whose parents had a higher education level showed significantly lower BLLs (father, *F* = 19.45; mother, *F* = 22.35; both *p* < 0.05). When the yearly household income was higher, the children's BLLs were lower; however, this difference was not statistically significant (*F* = 2.74; *p* > 0.05). There was a significant difference in the children's BLLs according to their parents' occupation: children whose parents had lead-related occupations showed significantly higher BLLs (father, *F* = 14.04; mother, *F* = 13.24; both *p* < 0.05). BLLs in outlanders were higher than those in local children (*F* = 8.06; *p* < 0.05). The BLLs of children from the Pengjie district were higher than that of the Fengjiang district, but the difference was not significant (*F* = 0.53; *p* > 0.05). Neither the type of drinking water (tap, ground, surface, or pure) nor passive smoking affected the children's BLLs (drinking water, *F* = 0.7; smoking, *F* = 2.14; both *p* > 0.05). The use of decorative tableware was significantly associated with elevated BLLs (*F* = 4.47; *p* < 0.05). Children who lived on higher residential floors showed significantly lower BLLs (*F* = 13.2; *p* < 0.05). The family-style small factory was not related to children' BLLs (*F* = 0.57; *p* > 0.05), although this may have been due to the fact that the child's residence was not in close proximity to this type of factory. On the other hand, when the factory was located close to the child's residence, BLLs were significantly elevated (*F* = 7.25; *p* < 0.05).

The risk factors associated with children's EBLLs are shown in Table 3. Their mother's level of education, their father's occupation, the use of decorative tableware, their exposure to makeup (i.e., kissing their mother who was wearing makeup on the cheek), and the residential floor on which the child lived were all risk factors for EBBLs (ORs of 1.42, 1.21, 1.11, 1.19, and 1.27, resp.). On the other hand, hand washing before eating food was a protective factor for EBLL (OR of 0.88).

## 4. Discussion

Lead poisoning still remains a major public health problem, with industrial pollution being one of the most important causes among children in China [3]. In an area of high lead pollution (Taizhou, China), we found that the BLLs of preschool children were 56.44 *μ*g/L on average, with 8.82% of children showing concentrations >100 *μ*g/L and 43.90% showing concentrations >50 *μ*g/L. These levels are higher than the average BLLs across the nation [23] and higher than those found in the United States [21].

Children who lived close to the lead-based factory had higher BLLs than those who lived further away (*T* = −3.05; *p* < 0.05). Similarly, other studies have shown that villages with gold ore-processing activity are at increased risk for childhood lead poisoning [22]. In another study, the lead industry was reported to cause serious environmental pollution that led to high BLLs in children living nearby [25].

We also found that boys in this region have higher BLLs than girls. This finding is consistent with previous studies in China, which reported that the BLLs in males and females (aged 0–18 years) were 48.8 *μ*g/L and 46.1 *μ*g/L, respectively [20, 23]. These gender-based differences may be related to the greater autonomous behavior and outdoor activities found in boys as they grow, leading to increased contact with environmental lead pollution. We also showed that BLLs varied with age, in agreement with previous studies, with the highest BLLs found in children aged 3–5 years [16, 23].

The parents' educational level was negatively correlated with children's BLLs in this study; that is, the higher parents' education was, the lower the BLLs were in their children. Earlier studies also found that parents with less than a high school degree more frequently had children with higher BLLs. This is because the parents' level of education is related to their learning and understanding of lead poisoning. Therefore, parents with higher education levels may correct bad habits in their children that may increase their exposure to lead, such as hand-to-mouth contact and biting nails.

In this study, we found that when parents' work in a lead-related occupation, their children's BLLs are higher than those whose parents' did not work in lead-related jobs. This is because parents whose work is lead-related could bring lead home, thereby exposing their children. Indeed, an earlier study showed that parents in a lead-related occupation were at risk of having children with increased BLLs, presumably due to lead dust carried from the workplace to their home [25–27]. Moreover, a recent review suggested that numerous environmental risk factors might be critical for lead exposure in children [2] and that worksites should adopt better containment facilities. As further evidence, improving workplace practices was shown to reduce BLLs among bridge painters in a previous study [28].

Hand washing was found to be a protective factor for BLLs in children in this study. As lead dust is inhaled and ingested via children's unwashed hands, hand washing can eliminate the digestive component of lead dust exposure [25]. On the other hand, the use of decorative tableware was found to be a risk factor for EBLLs. This is because the colorful material used for children's tableware often contains lead. When lead-containing tableware comes in contact with acidic food, lead can be dissolved, which can increase the lead level in the food. Indeed, an earlier study showed that using melamine plates was significantly correlated with higher BLLs in children in Bangladesh [29]. Similarly, in a case report of lead poisoning, the source of the lead was suspected to be the hand-painted mugs and small antique teaspoon that the patient used for his coffee [30]. Further testing revealed the spoon contained 50% lead and one of the mugs consisted of ~1% lead [30].

EBLLs were also related to children kissing their mother's cheeks when they were still wearing makeup. Lead has been used as cosmetic whitening agent since ancient times, as lead sulfate is white and stable in nature. As lipsticks are used daily, even minute amounts of lead in the lipstick could accumulate over time, resulting in lead poisoning. Therefore, cosmetics safety should be assessed not only by determining the presence of hazardous contents, but also by comparing estimated exposures with health-based standards. In fact, one study showed that lead was detected in 24 lipsticks (75%), with an average concentration of 0.36 ± 0.39 ppm [31]. Therefore, when lipstick is used at the estimated average daily rate, the estimated intakes were >20% [31]. Another study investigated the lead and cadmium contents in the most frequently used cosmetic product brands in Iran and found that the lead concentration in the lipsticks was within the range of 0.08–5.2 *μ*g/g [32]. Thus, the continuous use of these cosmetics can increase the absorption of heavy metals, especially cadmium and lead, which can be harmful, especially for children [32]. Lead and cadmium concentrations are also high in creams and lotions, with most white creams and white colored cosmetics containing higher levels of metal contaminants than those of others colors [33].

Our study also showed that floor of the residential building on which the children lived was a risk factor for EBLLs, with lower floors resulting in higher BLLs. This may be due to house dust, which has long been known to be a significant exposure source of lead, particularly for children. Factories close to residential areas can produce industrial pollutants and lead dust spills into nearby residential areas, contaminating the local residents. In addition, as the density of lead is greater than air, lead is suspended to be within the children's breathing zone, and these lead molecules are small enough to be absorbed through the child's lungs [25, 34]. Furthermore, a study in Japan indicated that the distribution of lead in house dust (i.e., the living environment) showed large variation, with a median concentration of 83.2 mg/kg and a geometric mean concentration of 97.8 mg/kg [35]. A meta-analysis about the risk factors of child lead poisoning in China showed that living on the ground floor was a strong risk factor for EBLLs in children [36].

To the best of our knowledge, this was the first study to examine the relationship between exposure risk factors and EBLLs in preschool children of Taizhou, a lead polluted area. Our findings appear to support the role of lead-based factories/workshops around the living area as a fundamental determinant for EBLLs among Taizhou children. However, there were some limitations to this study. First, the BLLs were determined at only one point in time, making it difficult to determine the degree to which our results reflect the circumstances of lead exposure throughout the whole period of childhood development. Second, we did not remeasure environmental lead levels after one year, so we could not assess the degree of persistence of lead contamination in the environment and link it to the retested BLLs.

We planned to understand the risk factors to lead in the various population studies conducted worldwide and also to correlate it with the current efforts in order to prevent adverse health effects.

In conclusion, we have identified a number of risk factors relating to EBLLs in preschool children in a lead polluted area in China. These risk factors should now be used to improve public health in these areas to prevent adverse health effects; for example, policies should be developed that ensure lead-based industries are not placed in close proximity to residential areas. Moreover, the reduction of lead concentrations in tableware and cosmetics and the promotion of hand washing could reduce BLLs in children.

## Figures and Tables

**Table 1:** Main characteristics of the preschool children included in the study.

Characteristics	*N* (%)
Sex	
Male	1087 (53.9%)
Female	931 (46.1%)
Age (years)	
<3	65 (3.2%)
3 to <4	294 (14.6%)
4 to <5	604 (29.9%)
5 to <6	670 (33.2%)
≥6	385 (19.1%)
Residence	
Outlander	765 (37.9%)
Native	1253 (62.1%)
Town	
Fengjiang	1264 (62.6%)
Pengjie	754 (37.4%)
Father's education	
Primary school	585 (29.0%)
Middle and high school	1335 (66.2%)
College or higher	98 (4.9%)
Father's occupation	
Lead-related	1033 (51.2%)
Not lead-related	985 (48.8%)
Mother's education	
Primary school	709 (35.1%)
Middle and high school	1197 (59.3%)
College or higher	112 (5.6%)
Mother's occupation	
Lead-related	926 (45.9%)
Not lead-related	1092 (54.1%)
Cares' education	
Primary school	877 (43.5%)
Middle and high school	1055 (52.3%)
College or higher	86 (4.3%)
Yearly household income (RMB)	
<10000	679 (33.7%)
10000–15000	977 (48.4%)
>15000	362 (17.9%)

**Table 2:** Main characteristics of preschool children with different BLLs.

Characteristics	BLLs (*μ*g/L)		*F* (*p* value)	BLL group			*χ* ^2^ (*p* value)
				<50 (*μ*g/L)	50–<100 (*μ*g/L)	≥100 (*μ*g/L)	
	Mean ± SD	Geometric mean		*N* (%)	*N* (%)	*N* (%)	
Sex							
Male	59.7 ± 41.2	51.5	25.94 (<0.0001)	571 (52.5)	396 (36.4)	120 (11.1)	19.71 (<0.0001)
Female	52.6 ± 34.6	46.0		561 (60.3)	312 (33.5)	58 (6.2)	
Age (years)							
<3	54.8 ± 57.2	44.7	2.88 (0.0215)	41 (62.1)	21 (31.8)	4 (6.1)	20.80 (0.0077)
3 to <4	62.8 ± 51.1	51.8		157 (53.4)	97 (33.0)	40 (13.6)	
4 to <5	56.9 ± 32.9	50.3		312 (51.7)	244 (40.4)	48 (7.9)	
5 to <6	54.3 ± 36.7	47.2		397 (59.3)	218 (32.5)	55 (8.2)	
≥6	54.8 ± 33.5	48.1		225 (58.6)	128 (33.3)	31 (8.1)	
Residence							
Outlander	57.7 ± 32.9	50.9	8.06 (0.0046)	387 (50.6)	310 (40.5)	68 (8.9)	17.05 (0.0002)
Native	55.7 ± 41.4	47.7		745 (59.5)	398 (31.8)	110 (8.7)	
Town							
Fengjiang	54.8 ± 30.4	48.6	0.53 (0.4653)	692 (54.8)	471 (37.3)	101 (7.9)	8.32 (0.0156)
Pengjie	59.2 ± 48.9	49.4		440 (58.4)	237 (31.4)	77 (10.2)	
Father's education							
Primary school	60.0 ± 35.0	53.0^*∗*^	19.45 (0.0005)	286 (48.9)	237 (40.5)	62 (10.6)	26.58 (<0.0001)
Middle and high school	55.75 ± 40.27	48.0^*∗*^		774 (58.0)	449 (33.6)	112 (8.4)	
College or higher	44.4 ± 28.2	38.8^*∗*^		72 (73.5)	22 (22.5)	4 (4.0)	
Father's occupation							
Lead related	59.0 ± 40.7	50.9	14.04 (0.0002)	545 (52.8)	387 (37.5)	101 (9.7)	9.81 (0.0074)
Not lead-related	53.8 ± 35.6	46.8		587 (59.6)	321 (32.6)	77 (7.8)	
Mother's education							
Primary school	60.3 ± 36.7	52.6^*∗*^	22.35 (<0.0001)	346 (48.8)	281 (39.6)	82 (11.6)	40.13 (<0.0001)
Middle and high school	55.3 ± 39.9	47.9^*∗*^		700 (58.5)	406 (33.9)	91 (7.6)	
College or higher	44.1 ± 29.5	38.2^*∗*^		86 (76.8)	21 (18.8)	5 (4.4)	
Mother's occupation							
Lead-related	59.5 ± 43.4	51.1	13.24 (0.0003)	486 (52.5)	347 (37.5)	93 (10.0)	9.66 (0.0080)
Not lead-related	53.8 ± 33.4	47.1		646 (59.2)	361 (33.2)	85 (7.6)	
Care giver's education							
Primary school	57.8 ± 34.4	50.8^#^	8.77 (0.0002)	452 (51.5)	341 (38.9)	84 (9.6)	21.46 (0.0003)
Middle and high school	56.0 ± 41.8	48.1^*∗*^		616 (58.4)	350 (33.2)	89 (8.4)	
College or higher	47.4 ± 32.8	40.8^*∗*#^		64 (74.4)	17 (19.8)	5 (5.8)	
Yearly household income (RMB)							
<10000	58.5 ± 43.0	50.2	2.74 (0.0649)	365 (53.8)	255 (37.6)	59 (8.7)	8.19 (0.0847)
10000–15000	55.8 ± 35.4	48.9		542 (55.5)	348 (35.6)	87 (8.9)	
>15000	54.3 ± 37.1	46.5		225 (62.2)	105 (29.0)	32 (8.8)	
Residential floor							
1st floor	60.5 ± 35.9	53.1^*∗*^	13.2 (<0.0001)	300 (47.8)	258 (41.1)	70 (11.1)	35.12 (<0.0001)
2nd to 3rd floor	56.4 ± 42.1	48.2^*∗*^		641 (58.3)	362 (32.9)	96 (8.7)	
4th to 6th floor	48.3 ± 25.9	43.1^*∗*^		181 (65.6)	83 (30.1)	12 (4.3)	
>6th floor	39.3 ± 11.1	37.7^*∗*^		10 (71.4)	4 (28.6)	0 (0)	
Family-style small factory							
No	56.6 ± 37.9	49.2	0.57 (0.4512)	749 (54.9)	493 (36.2)	121 (8.9)	1.19 (0.2334)
Yes	56.2 ± 39.5	48.3		383 (58.5)	215 (32.8)	57 (8.7)	
Passive smoking							
Never	58.9 ± 42.7	50.1	2.14 (0.0927)	416 (54.0)	277 (36.0)	77 (10.0)	5.872 (0.4376)
Once in a while	54.2 ± 36.1	47.4		522 (58.7)	299 (33.6)	68 (7.7)	
Often	57.2 ± 34.3	50.3		172 (53.6)	119 (37.1)	30 (9.4)	
Always	53.3 ± 27.8	47.3		22 (57.9)	13 (34.2)	3 (7.9)	
Use of decorative tableware							
Never	50.3 ± 30.5	50.6^*∗*^	4.47 (0.0039)	97 (63.4)	48 (31.4)	8 (5.2)	17.36 (0.0081)
Sometimes	52.9 ± 37.2	49.0		236 (58.4)	146 (36.1)	22 (5.5)	
Half of the time	55.7 ± 33.4	46.4^*∗*^		304 (55.6)	198 (36.2)	45 (8.2)	
Always	59.5 ± 42.5	44.9^*∗*^		495 (54.2)	316 (34.6)	103 (11.2)	
Drinking water							
Tap water	56.7 ± 39.3	48.9	0.70 (0.5529)	1047 (56.3)	648 (34.9)	164 (8.8)	2.38 (0.8815)
Ground water	55.0 ± 24.5	50.9		18 (54.5)	13 (39.4)	2 (6.1)	
Surface water	59.5 ± 31.3	53.7		13 (44.8)	12 (41.4)	4 (13.8)	
Pure water	51.5 ± 24.9	46.6		54 (55.7)	35 (36.1)	8 (8.2)	
Factory around the house or residential building							
Yes	58.4 ± 42.1	50.1	7.25 (0.0071)	670 (54.7)	435 (35.5)	121 (9.8)	5.20 (0.0742)
No	53.4 ± 31.6	47.1		462 (58.3)	273 (34.5)	57 (7.2)	

^*∗*, #^ *p* < 0.05 tested by Bonferroni for multiple comparison between any two groups in a variable.

**Table 3:** Factors affecting elevated blood lead levels in the children (logistic regression analysis).

Variable	*β*	Wald *χ*^2^	*p*	95% CI of OR
Sex	0.3266	12.8442	0.0003	1.386 (1.160–1.657)
Age	0.1075	6.5174	0.0107	1.113 (1.025–1.209)
Mother's education	0.3504	17.5834	<0.0001	1.420 (1.205–1.672)
Father's occupation	0.1903	4.4029	0.0359	1.210 (1.013–1.445)
Hand washing	−0.1272	5.5541	0.0184	0.881 (0.792–0.979)
Decorative tableware	0.1040	5.0479	0.0247	1.110 (1.013–1.215)
Makeup	0.1727	4.8991	0.0269	1.188 (1.020–1.385)
Residential floor	0.2420	11.7934	0.0006	1.274 (1.109–1.463)
